# Congenital Methemoglobinemia: First Confirmed Case in the Arab Population with a Novel Variant in the *CYB5R* Gene in the State of Qatar: A Case Report

**DOI:** 10.2147/JBM.S395865

**Published:** 2023-03-31

**Authors:** Abdulrahman Al-Abdulmalek, Reem Al-Sulaiman, Mohammad Abu-Tineh, Mohamed A Yassin

**Affiliations:** 1 Department of Internal Medicine, Hamad Medical Cooperation, Doha, Qatar; 2 Department of Medical Oncology/Hematology, National Center for Cancer Care and Research, Doha, Qatar

**Keywords:** methemoglobinemia, Arab, Qatar

## Abstract

Methemoglobinemia (MetHb) is a rare hematological condition characterized by high methemoglobin levels in the blood. It happens when hemoglobin is oxidized, resulting in hypoxia and cyanosis, which may occur in inherited or acquired forms. Inherited or congenital methemoglobinemia is a rare autosomal recessive condition and has never been reported in the Arab population. Here we report a case of a 22-year-old Arab man with a positive family history who presented with bluish discoloration of the fingers and lips and was found to have methemoglobinemia. Genetic study on the patient and his family revealed compound heterozygous variants in the CYB5R3 Exon 5 c.431G>A p.Gly144Asp likely pathogenic variant and CYB5R3 Exon 9 c.871G>A p.Val291Met variant of unknown significance. We suggest that the novel c.871G>A p.Val291Met variant could be causative for methemoglobinemia.

## Introduction

Methemoglobinemia (MetHb) is a rare hematological condition that occurs when hemoglobin is transformed into an oxidized form, resulting in a decrease in oxygen delivery to the tissues, causing hypoxia and cyanosis as common clinical manifestations.^1^,^2^ The symptoms’ severity is directly proportional to the methemoglobin level. As it increases, the symptoms can progress, and the patient can develop headaches, fatigue, dizziness, acidosis, arrhythmia, seizures, and coma (MetHb >50%) which may lead to death (>70%).^3^ Methemoglobinemia can be acquired due to offending agents such as drugs or may be inherited or congenital. Methemoglobinemia is rare and less common than the acquired form and is caused by cytochrome *b*_5_ reductase (CYB5R) deficiency and mutations in the *CYB5R3* gene. The *CYB5R3* gene encodes cytochrome *b*_5_ reductase-3, an enzyme that catalyzes the transfer of reducing equivalents from NADH to cytochrome *b*_5_. The soluble isoform of CYB5R3 is present in erythrocytes and significantly reduces methemoglobin to hemoglobin.^2^ Biallelic variants in CYB5R3 have been reported in association with autosomal recessive congenital methemoglobinemia type I and type II. Type I methemoglobinemia is characterized by mild symptoms of cyanosis, whereas type II has more severe symptoms, including neurological manifestation.^2^

In this case report, we report the first case of congenital methemoglobinemia in the Arab population, specifically in the State of Qatar, with heterozygous compound variants in CYB5R3, including a novel variant gene.

## Case Presentation

A 22-year-old Arab Middle Eastern Qatari man with no significant past medical history presented to the hematology clinic to evaluate the bluish discoloration of his lips and extremities. The patient denied using any drugs, topical creams, or exposure to any exogenous agents. The patient also denied recent surgeries.

**Figure 1:**
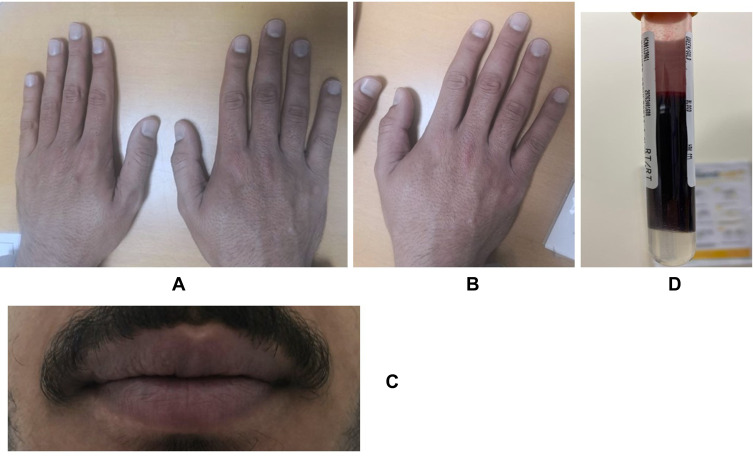
Showed cyanosis of the hands, fingers and lips(**A–C**). Also, it showed dark brown color blood (**D**). These finding are common manifestation of congenital methemoglobinemia.

On physical examination, he was not in respiratory distress. Cyanosis was noticeable on the hands, toes, and lips (Figure 1A). Vital signs were stable and included an arterial oxygen saturation of 94–96% on room air. His blood tests showed hemoglobin (Hb) of 16.3, a mean corpuscular volume (MCV) of 88.8 fl, and normal kidney and liver function.

When the blood sample was obtained, it was noticed that the color of the blood was dark brown (Figure 1D). The arterial blood gas was obtained, resulting in a pH of 7.39, pCO_2_ of 36.3 mmHg, and pO_2_ of 121 mmHg.

Due to the apparent cyanosis that is not corrected with oxygen supplementation and normal pO_2_, methemoglobinemia was suspected. Therefore, co-oximetry was performed on the same arterial blood sample, which showed a low FO_2_Hb of 77.4 and a high methemoglobin level of 20.8%.

The patient reported a family history of a similar condition in his sister, who was also evaluated in the hematology clinic and was found to have methemoglobinemia. Given the patient’s personal and family history, congenital methemoglobinemia was suspected. The patient and his sister were referred to the genetics clinic to rule out congenital methemoglobinemia in this family.

**Figure 2:**
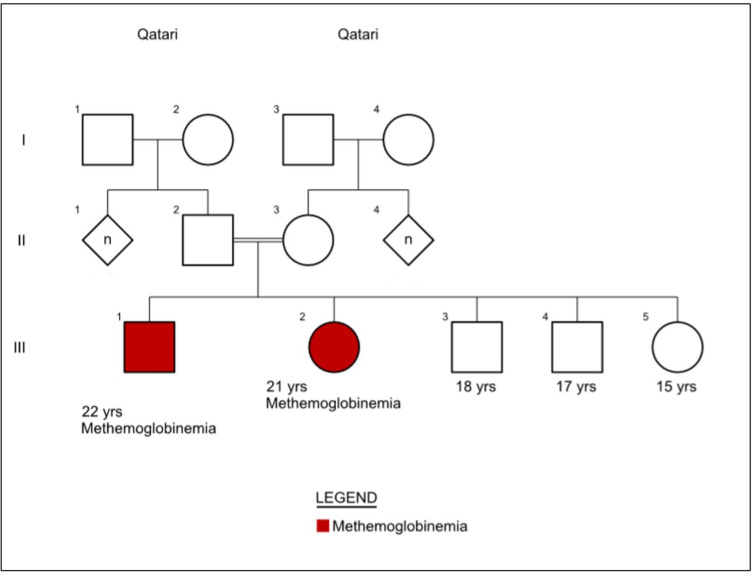
Autosomal recessive pedigrees tend to show fewer affected individuals and are often described as “skipping” generations. Thus, the primary feature distinguishing autosomal recessive from dominantly inherited traits is that unaffected individuals can have affected offspring, as seen by the red-colored affected children, from carrier parents from the second generation.

Family history revealed that the siblings are the product of consanguineous marriage (parents are first cousins), and no cases of methemoglobinemia or similar illnesses were reported in the family except for the two siblings, suggesting an autosomal recessive inheritance in this family given the consanguinity and the horizontal inheritance (Figure 2).

The Whole Exome Sequencing (WES) study was done on both siblings, including their mother. Both siblings carried heterozygous compound variants in the *CYB5R3* gene as follows:

First, CYB5R3 Exon 5 c.431G > A p.Gly144Asp-likely pathogenic heterozygous variant (inherited from the mother). This variant was reported in the literature and was observed in a homozygous state in individuals with methemoglobinemia type I.^4^ Both the mother and the affected sibling are heterozygous for this variant.

The second variant is CYB5R3 Exon 9 c.871G > A p.Val291Met-Variant of uncertain significance. This variant has not been previously published as pathogenic or benign. There are not enough data in large population cohorts to assess the frequency of this variant in publicly available databases.

Both siblings are carriers of this variant. However, the mother is not a carrier. This variant might have been inherited from the father. Neither the father nor the unaffected siblings could segregate this variant for its significance. Given that both affected siblings are carriers of this VUS, it is suggestive that this VUS might contribute to their phenotype and the likely pathogenic heterozygous variant.

A diagnosis of cytochrome *b*_5_ reductase (CYB5R) deficiency was made, and the patient was treated with Vitamin C 500 mg daily. The patient showed significant improvement, evidenced by the reduced MetHb level and resolution of his cyanosis.

## Discussion

Hemoglobin consists of four atoms of iron bound in a complex quaternary structure of proteins. In a normal hemoglobin molecule, iron is found in the ferrous state (Fe^2+^), which facilitates the binding and delivery of oxygen. Methemoglobin is a condition wherein the iron exists in an oxidized state (Fe^3+^) which has a reduced ability to bind to oxygen.^4^,^5^, Iron in the Fe^3+^ state affects the remaining ferrous ions (Fe^2+^) to increase affinity for binding oxygen, resulting in impaired delivery of oxygen to the tissues. As a result, the oxygen–hemoglobin dissociation curve will be shifted to the left^5–7^

Methemoglobin forms as a result of oxidative stress and can form 0.5–3% per day, which is regulated by several counter-regulatory mechanisms to maintain a level of methemoglobin of less than 1%.^4^ A major pathway for the reduction of methemoglobin back to hemoglobin is facilitated by the cytochrome *b*_5_ reductase pathway, which is responsible for at least 95% of the methemoglobin reduction to hemoglobin. Another method is via the NAPH-methemoglobin reductase pathway, which exists as an alternative, accounting for 5%.^7^ Although the NADPH-reductase pathway is the responsible minor pathway, an exogenous pharmacological agent such as methylene blue can enhance it.^2^,^5^,^8^

Two main ways may lead to increased methemoglobin levels. Firstly, a consequence of exposure to oxidizing agents, drugs, or metabolites causes the conversion of hemoglobin to methemoglobin to be increased by up to 1000-fold, overwhelming the regulatory mechanisms.^1^ One oxidizing agent includes contaminated water, pharmaceutical drugs such as antimalarial drugs, dapsone, nitrites, and local anesthetic drugs such as lidocaine and benzocaine.^1^,^3^ Secondly, methemoglobin can be caused by genetic mutations that lead to intrinsic enzyme deficiencies in protective mechanisms, or the presence of abnormally structured hemoglobin M. These can be categorized into acquired and congenital (inherited) causes of methemoglobinemia, respectively.^2^,^3^

This paper will focus on congenital causes. There are three kinds of hereditary methemoglobinemia, the most common being (CYB5R) deficiency. Less commonly reported are cytochrome *b*_5_ deficiency and hemoglobin M disease. In hemoglobin M disease, an amino acid substitution mutation occurs, wherein tyrosine residues in the heme pocket replace histidine. In turn, heme-bound iron interacts with tyrosine phenolate sidechains and becomes resistant to reduction.^10–12^

Cytochrome *b*_5_ reductases are a group of essential enzymes present in all tissues; there are four known *CYB5R* genes that code for this protein, measuring approximately 32 kilobases in length and located on chromosome 22q 13-qter.^2^ Mutations usually cause cytochrome *b*_5_ reductase deficiency in one of the four *CYB5R* genes, which are usually inherited in an autosomal recessive fashion. Two types of CYB5R deficiency are categorized as type I and type II. In type I, the enzyme deficiency is restricted to erythrocytes; consequently, patients may present with asymptomatic cyanosis or mild symptoms.^2^ Type II is much rarer, accounting for less than 10% of all cases of hereditary methemoglobinemia. This condition features a loss of CYB5R in all cells as opposed to type I. Patients generally have more severe manifestations of type II, such as neurological dysfunction and shortened life expectancy. Individuals with heterozygous variants in the *CYB5R3* gene are asymptomatic as they have some functional enzymes.^2^,^6^ However, they are at a higher risk of developing an acute presentation of clinically significant methemoglobinemia upon exposure to oxidizing agents.^2^ However, phenotypical symptoms are usually present in individuals with homozygous variants.^9^

The distribution of the disorder is global; however, endemic zones exist. For example, the type I variant is reported more frequently in Athabascan Alaskans, Alaskan Eskimos,^11^ Navajo Indians,^13^,^14^ Puerto Rico, and Yakutsk natives of Siberia.^14^

Cytochrome *b*_5_ reductase deficiency is a rare disorder in the Arab population, with only one reported case in the literature.^15^ However, the case was diagnosed according to clinical presentation, measured methemoglobin levels, and cytochrome *b*_5_ reductase enzyme activity. There have not been any reports in the literature of genetics-based diagnoses of this condition in the Arab population. Here, we are reporting the first case of type I Cytochrome *b*_5_ reductase deficiency confirmed by genetic testing.

## Conclusion

From the genetic study, it is suggested that the identified compound heterozygous variants in the *CYB5R3* gene might be causative of the congenital methemoglobinemia in our patients (most likely methemoglobinemia type I), supported by the clinical phenotype of the patients, the reported consanguinity in the family, and the autosomal recessive inheritance pattern. Future family segregation for the CYB5R3 c.871G>A p.Val291Met variant (especially in newly affected cases) to better address its significance when it is inherited with the likely pathogenic variant c.431G>A p.Gly144Asp.

## Abbreviations

MetHb, methemoglobinemia; CYB5R, cytochrome *b*_5_ reductase; WES, whole exome sequencing; NADH, nicotinamide adenine dinucleotide; NADPH, nicotinamide adenine dinucleotide phosphate oxidase; Hb, hemoglobin; MCV, mean corpuscular volume.
